# Interface Strength, Damage and Fracture between Ceramic Films and Metallic Substrates

**DOI:** 10.3390/ma14020353

**Published:** 2021-01-12

**Authors:** Lihong Liang, Linfeng Chen, Luobing Wu, Huifeng Tan

**Affiliations:** 1 Beijing Key Lab of Health Monitoring and Self-Recovery for High-End Mechanical Equipment, College of Mechanical and Electrical Engineering, Beijing University of Chemical Technology, Beijing 100029, China; 2020200591@mail.buct.edu.cn (L.C.); binggewu@foxmail.com (L.W.); 2 National Key Laboratory of Science and Technology on Advanced Composites in Special Environments, Harbin Institute of Technology, Harbin 150001, Heilongjiang, China; tanhf@hit.edu.cn

**Keywords:** mechanical properties, modelling, ceramic films, interface strength, fracture energy

## Abstract

Interface strength, damage and fracture properties between ceramic films and metallic substrates affect the service reliability of related parts. The films’ thickness, grain size and residual stress affect the interface properties and fracture behavior, thus related studies attract great attention. In this paper, the interface damage evolution and fracture behavior between ceramic films and metallic substrates were simulated by developing a three dimensional finite element model of alumina films on Ni substrates with cohesive elements in the interfaces. The interface fracture energy as a key parameter in the simulation was firstly determined based on its thermodynamic definition. The simulation results show the Mises stress distribution and damage evolution of the film/substrate structures during uniaxial tensile loading. Specially, when grain size of the films is in nanoscale, the interface strength increases obviously, agreeing with the previous experimental results. The effects of residual stress on interface properties was further simulated. The interface strength was found to decrease with increasing radial residual force and the axial residual pressure increases the interface strength. When the thickness of the films increases, the interface strength keeps a constant but the speed of interface damage becomes faster, that is, the thicker films show catastrophic fracture. The underlying mechanism of damage speed was analyzed. Understanding these size effects and the effects of residual stress is helpful to guide the design of related parts.

## 1. Introduction

Interfaces between ceramic films and metallic substrates have wide application, such as the interface between thermal growth alumina layer and nickel alloy substrate in serviced turbine blades of aeronautical engines [1], ceramic-metal joints in electronic components [2] and so forth. Interface strength and fracture characteristics affect mechanical properties and service stability of related parts. Composition and defect effects on interface bonding properties have been studied, the deleterious effect of sulfur on interface adherence of thermal growth oxidation on bond coat was observed [3], sulfur segregation did not occur on defect-free interface. The effect of Nb and Ti on interface properties of Al/Al_2_O_3_ joints was analyzed, Nb and Ti improved wettability of alumina by Al at the high temperature [4]. The effect of impurity on alumina-niobium interface was studied, too [5]. On the other hand, residual thermal stress induced by fabrication of films affects interface properties and crack initiation, which has been analyzed and interface strength was found to increase with increasing residual compressive stress [6]. The stability of residual stress in alumina films and its effect on mechanical properties was once studied [7]. The influence of residual thermal stresses arising during processing of ceramics-metal joints was summarized adequately [8]. Note that size effect and thickness effect of ceramic films also influence interface properties, which has to be studied further yet. The report showed that the grain scale and microtexture of alumina affected its thermal expansion coefficient, which changed the interface structure and properties [9]. The smaller grain size and textured grain of alumina was found to reduce residual stress and inhibit cracking [10]. The steel specimen with apparent textured grains showed the poor cracking resistance and the fracture mode was improved by the heat treatment [11]. Recently, the effects of the thickness of the film on the crack density in the film and the crack initiation strain were investigated based on a residual stress model [12]. The effects of the specimen thickness and crack length on the variation of J integral were obtained by three dimensional finite element simulation [13]. The fracture mode was also found to change from coating cracking to interface cracking with increasing coating thickness [14]. Moreover, the detailed damage evolution affects interface fracture characteristics and attracts great attention. For example, tensile damage behavior of a sandwiched coating system was studied and a crack evolution mode of initiation, multiplication and saturation with increasing strain was proposed [15]. Damage kinetics of thermal barrier coatings with thermal cycling oxidation time was analyzed by acoustic emission method [16]. Damage models based on the Taylor’s extension and energy analysis were developed to describe coating cracking and failure behavior [17,18]. How the crack modes in films depend on material properties and thickness ratio was studied by numerically analysis based on energy release rate [19]. Cracking evolution in coatings under three-point loading has been simulated based on two dimensional finite element method and damage process can be observed digitally [14]. Three dimensional (3D) stress analysis during interface fracture of film/substrate structure is more desired to capture damage evolution characteristics.

In order to understand interface damage behavior and fracture properties deeply, interface fracture energy and its size effect was theoretically calculated firstly in this paper. Then 3D finite element models of alumina films on nickel substrates was established by inserting interface cohesive elements into the interfaces with corresponding fracture energy. The tension load vertical to the interfaces was applied on the films to simulate interface fracture between the films and substrates. The Mises stress evolution and interface damage characteristics was analyzed, the film thickness effect on the damage speed was revealed. Moreover, the fracture properties of nanocrystalline films and conventional films with grains in micron scale were compared to check the size effect. And the various effects of residual stress on interface strength was discovered.

## 2. Interface Fracture Energy Between Alumina Films and Ni Substrates

According to general thermodynamics of interface fracture, the intrinsic fracture energy *Γ* is the energy difference after and before the fracture of the interface, for interfaces between alumina films and nickel substrates

*Γ* = *γ_f_* + *γ_s_* − *γ_i_*, (1)

with surface energy *γ_f_* and *γ_s_* of the films and the substrates, respectively and interface free energy *γ_i_* between both. The surface energy of Ni can be obtained easily from many references *γ_s_* = 2.42 J/m^2^ [18,20], the surface energy of Al_2_O_3_ can be calculated based on the expression of surface energy [21]

(2) γf=Ub22−zszb−zszb12,

with the cohesive energy *U_b_* of Al_2_O_3_, the nearest neighbor atomic number *z_s_* and *z_b_* of surface atoms and bulk atoms, respectively. Considering the crystal structure of α-Al_2_O_3_ with densely hexagonal stacked oxygen ions and interstitial aluminum ions [22], average *z_s_* = 9 and *z_b_* = 12 were taken. *U_b_* = 147 eV was estimated based on the melting enthalpy of the alumina [23]. Therefore, the surface energy of alumina *γ_fc_* = 10.08 J/m^2^ with the subscript c represents conventional films with grains in micron scale. Furthermore, the size effect of surface energy has been studied for nanocrystals and is obvious for ceramic oxides [24]. The size-dependent surface energy of nanocrystalline alumina films *γ_fn_* can be expressed as

(3) $$\gamma_{fn}=\gamma_{fc}\left[1-\frac{1}{\left(d/d_{c}\right)-1}\right]\exp\left\{-\frac{2S_{c}}{3R\left[\left(d/d_{c}\right)-1\right]}\right\},$$

where *d* is average grain diameter of the films, 70 nm was considered here referring to the previous reports [24]. *d_c_* = *h/2* is a critical size of the corresponding crystal related to the bond length *h* (average 0.301 nm for Al_2_O_3_ [22]). *S_c_* = *U_b_*/*T_b_* is the sublimation entropy of the crystal and can be calculated by *E_b_* and the boiling point *T_b_* (3253.15 K for Al_2_O_3_ [25]). *R* is the ideal gas constant. According to Equation (3), the surface energy of nanocrystalline alumina films decreases about one time compared to that of the conventional ones, *γ_fn_* = 0.5 *γ_fc_* = 5.04 J/m^2^. When the grain size is larger 400 nm, the size effect can be neglected.

Interface free energy between two materials has been studied [24,26] and can be expressed as

(4) $$\gamma_{i}=\frac{2}{3R}\left[\frac{h_{f}S_{mf}H_{mf}}{V_{f}}+\frac{h_{s}S_{ms}H_{ms}}{V_{s}}\right],$$

with the melting entropy *S_m_*, the melting enthalpy *H_m_* and the molar volume *V*, the subscripts f and s represent the films and substrates, respectively. The corresponding parameters are in Table 1. According to the above expression, the interface free energy between alumina films and Ni substrates is *γ_i_* = 2.91 J/m^2^, close to the reported result [8]. Finally, the interface fracture energy *Γ* = 9.59 J/m^2^ for conventional films and *Γ* = 4.55 J/m^2^ for nanocrystalline films can be obtained based on Equation (1). Note that grain anisotropy affects interface fracture properties [10,11], simple size effect of isotropic grains was calculated here. If the grain shape factor was considered, the fracture energy of nanocrystalline films may be different with different surface energy according to Equation (1). On the other hand, residual thermal stress induced in the fabrication will also affect interface properties and damage behavior as above mentioned [6]. The following finite element model and interface fracture simulation were based on the calculated interface fracture energy here.

## 3. Finite Element Model of Alumina Film on Ni Substrate with Interface Cohesive Zone

**Figure 1:**
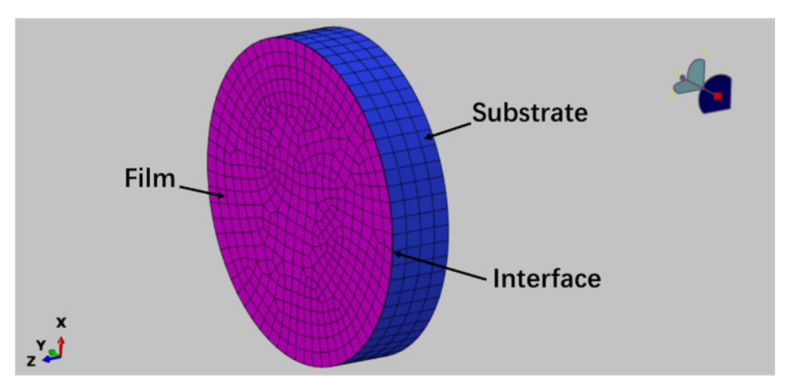
3D finite element model of film/substrate structure: the left end surface is alumina thin film and the right four layers mesh is the substrate, a cohesive element is inserted into the interface. The mesh division at the boundary is denser.

A three dimensional alumina film/Ni substrate model was established with the commercially available finite element code ABQUAS as shown in Figure 1, the substrate remains 2 mm thickness, the film thickness changes from micron scale to nanoscale (2 μm in Figure 1) in order to study film thickness effect and the zero thickness cohesive element was set at the interface between the film and the substrate. The hexahedral mesh was used and the total mesh number is 2892, among them there is 482 meshes in each layer and in the interface. The calculated result is mesh insensitive after the optimization of mesh. Note that the advancing front method was applied in mesh division, it is easy to realize a transition from coarse mesh to fine mesh. The substrate was fixed and constrained in six directions, the displacement load was applied on the film in direction vertical to the interface and the displacement in every step is 10^−5^ μm. The film was also constrained except in the loading direction. The elastic constitutive was assumed simply and the elements C3D8R was selected for both the film and the substrate and the cohesive element COH3D8 based on the bilinear constitutive was inserted into the interface. The elastic modulus of Al_2_O_3_ film and Ni substrate were 375 GPa and 200 GPa [24], respectively and Poisson ratio of the film and substrate were 0.291 and 0.3, respectively. Note that the substrate is elastic-plastic actually and the elastic-plastic constitutive was also considered, there was no influence on the results here since the yield strength of the substrate (800 MPa [14]) is much larger than the interface fracture strength (45.5 MPa, see the next section) and the deformation of the substrate is very small (<0.05%) when the interface fracture occurs. 

**Figure 2:**
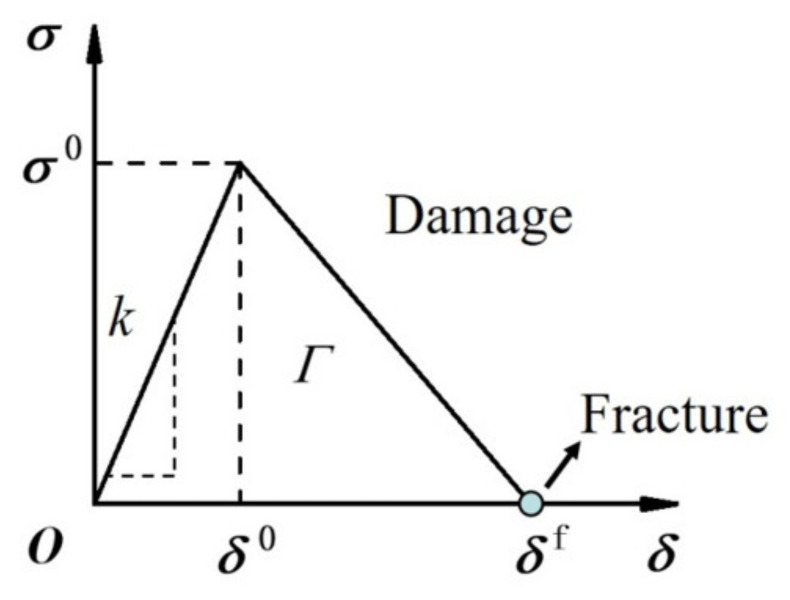
Interfacial stress *σ*-displacement *δ* relation with interface strength *σ*^0^, toughness *Γ*, initial stiffness *k*, damage displacement *δ*^0^ and fracture displacement *δ^f^*.

Interface cohesive element is based on the bilinear cohesive zone model [29], describing the relationship between the separation stress *σ* and the displacement *δ* of the interfacial atoms, as shown in Figure 2, the interfacial fracture energy *Γ* (i.e., intrinsic fracture toughness corresponding to the area under the *σ*-*δ* curve) and the interface strength *σ*^0^ (peak stress approximately) are two important parameters [13]. Interface stress-displacement relation is expressed as

(5) $$\sigma_{i}=\left\{\begin{array}{l} k_{i}\delta_{i},\\ \frac{\delta_{i}^{f}-\delta_{i}}{\delta_{i}^{f}-\delta_{i}^{0}}\sigma_{i}^{0}=\\ 0, \end{array}\left(1-D_{i}\right)k_{i}\delta_{i}\right.,\;\;\;\begin{array}{c} \delta_{i}<\delta_{i}^{0}\\ \delta_{i}^{0}\leq \delta_{i}<\delta_{i}^{f}\\ \delta_{i}\geq \delta_{i}^{f} \end{array},$$

where subscript *i* represent normal or tangent direction, *k* is interface initial stiffness and 2 × 10^6^ MPa/mm was taken considering that influence of cohesive element on material properties should be as small as possible and at the same time, *k* should be as large as possible for thin cohesive element [30]. For example, when a cohesive element was inserted into the interface between two layers of the substrate material with the modulus *E_s_*, it should not change the elastic modulus of the substrate, *k_s_t_s_* = *E_s_* with the substrate thickness *t_s_*. *k* is one order larger than the substrate stiffness *k_s_* and was assumed same in normal and tangent directions here. *δ*^0^ is the interface damage initial displacement corresponding to the strength *σ*^0^ and *δ^f^* is the fracture opening displacement at which *σ* decreases to be zero. *D* is damage variable. In Equation (5), the interface strength *σ*^0^ as an important interface quantity can be obtained by another important interface parameter- the fracture energy *Γ* combining with the fracture displacement *δ^f^* as shown in Figure 2. And the damage displacement *δ*^0^ can be determined by the initial stiffness *k* and *σ*^0^, *δ*^0^ is about one over ten of *δ^f^* here. 

After the interface stress reaches the strength value, interface damage initiates and the stress begins to decrease up to zero, interface fracture occurs. Damage variable *D* is expressed as

(6) $$D_{i}=\left\{\begin{array}{l} 0,\\ \frac{\delta_{i}^{f}\left(\delta_{i}^{f}-\delta_{i}\right)}{\delta_{i}(\delta_{i}^{f}-\delta_{i}^{0})}\\ 1, \end{array}\right.,\;\;\;\begin{array}{c} \delta_{i}<\delta_{i}^{0}\\ \delta_{i}^{0}\leq \delta_{i}<\delta_{i}^{f}\\ \delta_{i}\geq \delta_{i}^{f} \end{array}.$$

The quadratic nominal stress criterion was adopted for damage initiation as usual [31]. The interface fracture toughness is expressed as

(7) $${}_{\mathrm{\Gamma i}}=\int_{0}^{\delta {}_{f}}\sigma_{i}\left(\delta_{i}\right)d\delta_{i}.$$

When the work done by the traction *G* reaches the fracture toughness $\frac{G}{\Gamma }=1$, interface fracture occurs. Note that only the normal direction was considered simply here. The viscosity coefficient was taken as 0.0001 considering the convergence of cohesive element.

In this simulation, *Γ* is 4.55 J/m^2^ and 9.59 J/m^2^ for nanocrystalline films with average grain diameter 70 nm and conventional coarse-grain (diameter larger than 400 nm) films, respectively based on the interface fracture energy calculation in above section. The fracture energy is in the value range of J integral based on the similar finite element simulation of Al film’s peeling from alumina substrate [13]. And the interface strength *σ*^0^ and the corresponding displacement *δ*^0^ can be obtained at the given fracture displacement *δ^f^* since *Γ* is known according to the area of triangle curves showed in Figure 2. By combining Section 2 with Section 3, the microstructure effect of films on interface fracture was reflected by changing interface fracture energy in the cohesive element. At the same time, macro damage and fracture behavior of film/substrate structures can be simulated by the finite element method with the interface cohesive element.

## 4. Simulation Results and Discussion

### 4.1. Stress and Interface Damage Evolution

**Figure 3:**
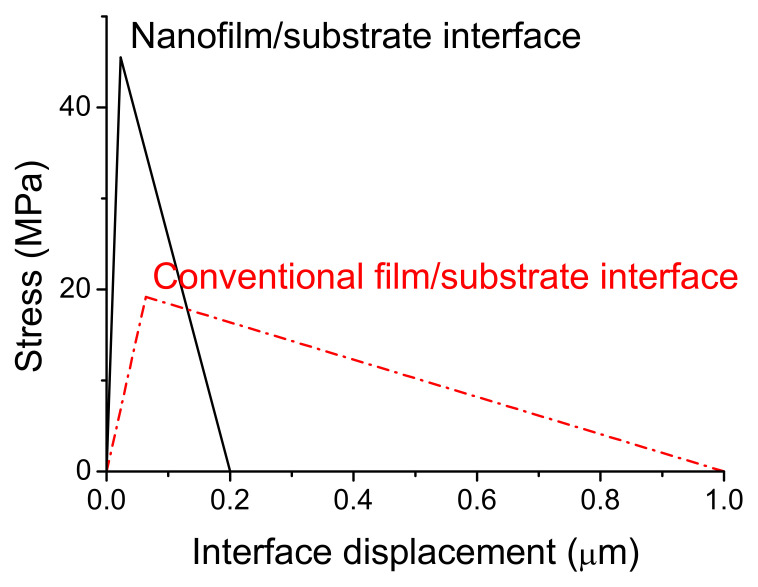
Interface stress-displacement relations of nanocrystalline ceramic film/substrate (black solid curve) and conventional thin film/substrate (red).

Nanocrystalline films was considered firstly based on advanced preparation technology [32], the interface fracture energy of 4.55 J/m^2^ was taken based on the calculation in Section 2 and the fracture displacement was assumed as 200 nm corresponding to several times of the grain size [24], then the interface strength can be obtained to be about 45.5 MPa, as showed by the peak stress of interface stress-displacement curve (black curve) in Figure 3. Note that the film and the substrate was considered as rigid firstly in Figure 3, the interface displacement equals to loading displacement.

**Figure 4:**
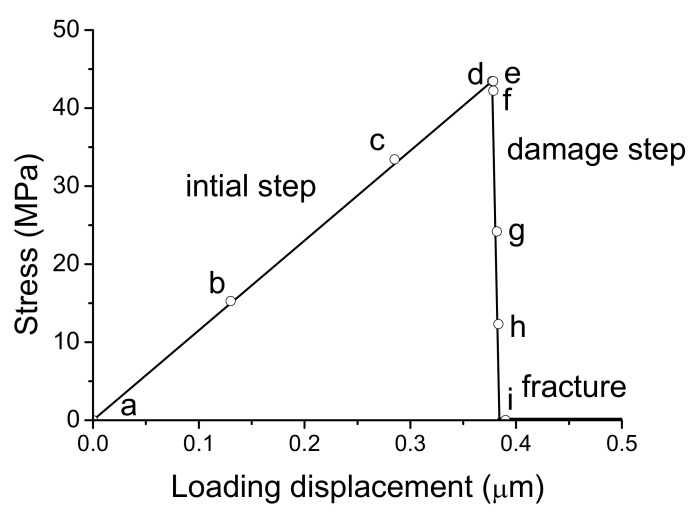
The total tensile stress-displacement relation of nanocrystalline film/substrate structure. The Mises stress evolution corresponding to a-i points in the curve were showed in Figure 5.

When elastic film and substrate were considered, the total tensile stress-displacement relation was obtained as shown in Figure 4. Note that the interface tensile stress is same to that in the film and substrate based on the series model [33] but the total displacement, that is, loading displacement includes the interface displacement and the displacements in the film and the substrate. Therefore, when interface fracture occurs, the total loading displacement is larger than the interface displacement and the total stiffness is smaller than the interface stiffness.

**Figure 5:**
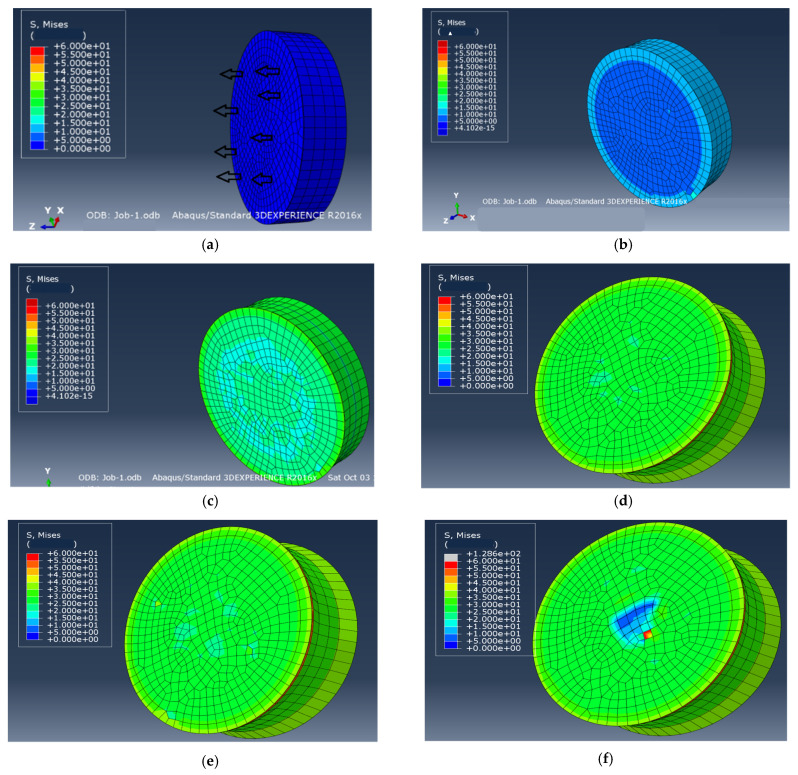

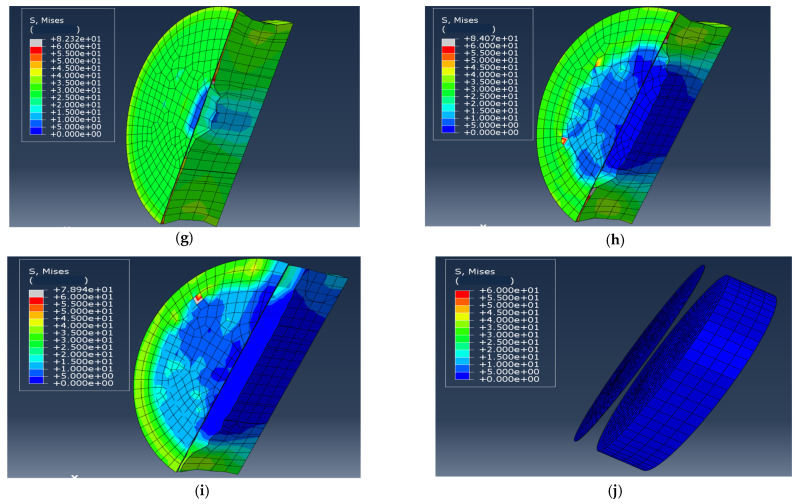
Mises stress evolution of the film/substrate structure under uniaxial tension with displacement loading from initial state (**a**) to complete interface fracture (**i**). (a) Mises stress at loading displacement 0, (**b**) at displacement 0.1302 μm, (**c**) at displacement 0.2852 μm, the stress increases continuously and becomes homogenous, the circumference stress is larger firstly, (**d**) at displacement 0.3774 μm, the interface stress reaches the maximum value, (**e**) at displacement 0.3784 μm after reaching the strength, the film stress begins to decrease, (**f**) at displacement 0.3787 μm, the zone of stress drop localizes at the central firstly and through the substrate, the interface crack begins from the central (frontal, (**g**) cross-section), (**h**) at displacement 0.3819 μm, the zone of stress drop extends from the central to the circumference, (**i**) at displacement 0.3834 μm, the interface fracture is mostly complete, (**j**) complete separation between the film and substrate. unit of stress: MPa.

The corresponding Mises stress evolution during loading was showed in Figure 5a–i. It can be seen that stress increases with loading in initial step and the circumference stress is larger firstly as shown in Figure 5b and the stress becomes homogeneous continuously with increasing displacement as showed in Figure 5c. When the stress reaches the strength value, the interface stress is maximum as shown in Figure 5d. After reaching the peak stress, the stress of the film begins to release as shown in Figure 5e and localizes in the central zone accompanied with the stress release of the interface and the substrate as showed in Figure 5f, which indicates the initiation of interface damage. Continuously, the zone of stress drop extends from the central zone to the periphery as showed in Figure 5g–h, up to complete fracture between the film and the substrate as showed in Figure 5i and the stress releases completely. The stress evolution in damage step shows the damage localization and crack propagation speed are fast with small increase of displacement, the damage detail were captured here. Note that ceramic film was assumed to be homogeneous in the simulation here. When microstructure inhomogeneity or residual stress exists in real parts, damage initiation and evolution should depend on corresponding condition. For example, residual thermal stress in the fabrication may induce initial defect in the boundary, the damage and cracking will initiate from there.

### 4.2. Increased Interface Strength of Nanocrystalline Films and the Effects of Residual Stress on Interface Strength 

When the conventional films are considered, the stress evolution characteristic is similar but the interface properties are different, the interface fracture energy increases about 2 times as calculated in Section 2 and the fracture displacement also increases (micron scale generally [24]) corresponding to increased microstructure scale. In order to compare the interface fracture strength of nanocrystalline films with that of conventional ones, interface fracture between a conventional coarse-grain film and the same substrate was also simulated. In the simulation, the interface fracture energy was taken as 9.59 J/m^2^ according to Equation (1), the fracture displacement was considered as 1 μm, the interface strength can be obtained as 19.18 MPa as showed in Figure 3 and the initial stiffness decreases correspondingly with increased interface damage displacement. It can be seen that the interface strength of nanocrystalline film increases about 2.4 times compared to the that of conventional film, agreeing with the previous experimental results [24]. The experimental measured average interface strength between nanostructured ZrO_2_ coatings and Ni alloy substrates is about 52 MPa, which increases about 2 times compared to 28 MPa for conventional coatings in the same tensile tests [24] (see Figure 4 in Ref. [24]).

On the other hand, the residual stress is induced usually when the temperature of film/substrate structures changes from a high temperature to the room temperature in the fabrication or from the room temperature to a high temperature in the service, the ceramic film is in compressive state due to its smaller thermal expansion coefficient compared to that of the metallic substrate during temperature drop and the substrate is in tensile state, vice versa. According to a simple estimation of interface thermal mismatch strain, the residual thermal stress can be expressed as

(8) $$\sigma_{r}=∆\alpha ∆T\frac{E_{f}E_{s}}{E_{f}+E_{s}}$$

where the difference of the thermal expansion coefficient is about (11.5−8) × 10^−6^/K= 3.5 × 10^−6^/K [8] with thermal expansion coefficient of Ni being 11.5 × 10^−6^/K and that of Al_2_O_3_ being 8 × 10^−6^/K, the elastic modulus of the film *E_f_* is 375 GPa and the elastic modulus of the substrate *E_s_* is 200 GPa, when the temperature difference Δ*T* = 1000 K is taken, the residual stress of 456.4 MPa can be obtained based on Equation (8). Actually, the stress is relaxed by the deformation of the substrate and the cracking of the film [34], the residual stress may be small. However, the residual stress may affect interface properties importantly.

**Figure 6:**
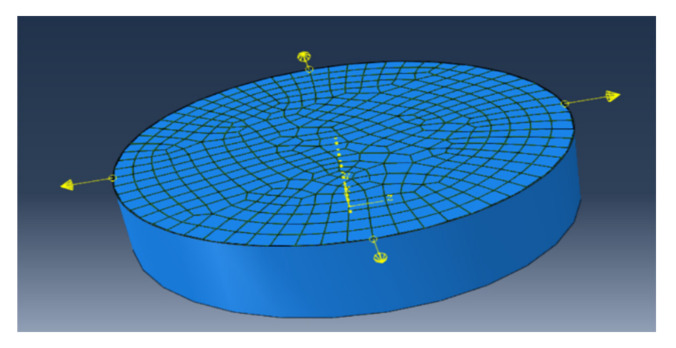
The residual force was applied in the nodes of elements of periphery of the interface before loading to simulate residual stress effect.

**Figure 7:**
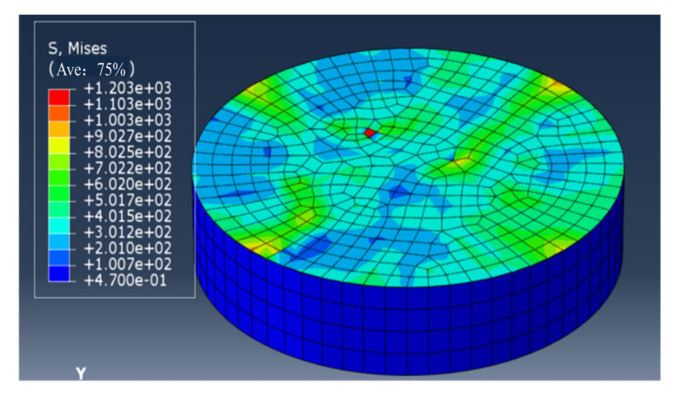
Mises stress distribution in the interface when the interface was applied 7 N tensile force at four nodes.

In order to simulate residual stress influence, the tensile force of 3 N, 5 N, 7 N and 10 N was respectively applied at approximately symmetric four nodes of the elements of the periphery of the interface along the radial direction as showed in Figure 6 (the initial force was also applied at eight nodes, the results is insensitive except for 10 N), then the same displacement loading vertical to the interface was applied to the film surface as showed in Figure 5a. The Mises stress of about 800 MPa was induced near the nodes naturally before loading as showed in Figure 7 and the stress in low stress zone is about 100 MPa. The average residual stress of 450 MPa induced in plane, as showed in Figure 7, is similar to the calculation result based on Equation (8). Even the residual stress of larger 1 GPa is induced in the local element, simulating real case with heterogeneous residual stress.

**Figure 8:**
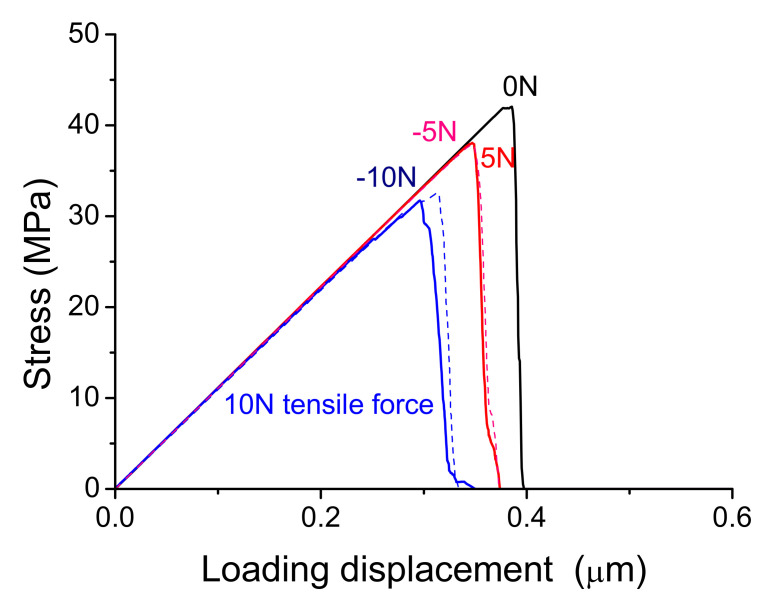
Effect of residual force on interface strength of the interfaces with residual tensile force of 10 N (blue solid curve) and 5 N (red solid curve), the black solid curve represents the result without residual force and the dashed navy and pink curves correspond to the results with residual compressive forces.

Figure 8 shows the interface strength (peak value of the stress) decreases with increasing residual force whether the force is in tensile (positive values of the force) or in compressive (the negative values) state. It can also be seen when the residual tensile and compressive forces of 5 N and -5N was applied, the interface strength decreases similarly. But when the residual force is larger than about 7 N, the tensile force induces larger decrease of the interface strength compared to the compressive one as showed in Figure 8 (10 N). When the large residual force was applied at the more nodes, the interface strength decreases further. Different from the central damage initiation of ideal interface without residual stress in above section, the damage starts from the stress concentration zone at the periphery of the interface.

**Figure 9:**
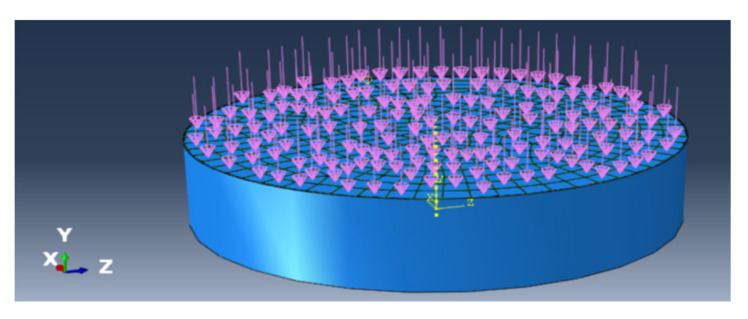
The residual pressure was applied on the top surface of the interface elements before loading.

**Figure 10:**
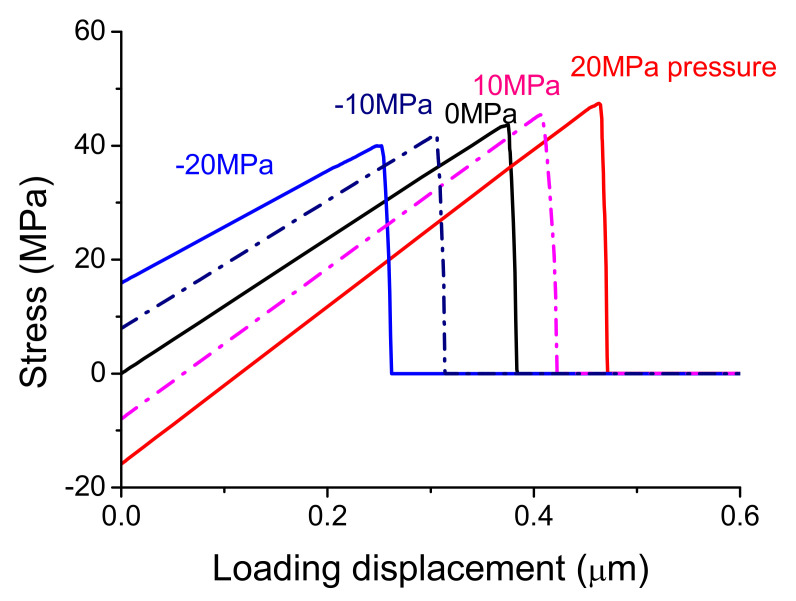
The residual pressure effects on the interface strength (peak stress) at tensile loading.

On the other hand, when the residual pressure of 10 MPa and 20 MPa, vertical to the interface, was respectively applied on the surface of the interface elements before loading as showed in Figure 9, the effects of residual stress out of plane was simulated further by the same tensile loading as showed in Figure 5a. Figure 10 shows when the initial stress is in compressive stress, the interface strength increases (red and pink curves) compared to that of interface without residual pressure (black curve). When the pressure was taken as the negative values and the initial stress is tensile stress, the interface strength decreases (blue and navy curves). As the residual pressure increases, the change of interface strength increases. Furthermore, when the residual stress out of plane and in plane were applied at the same time, the similar effects can be found. Certainly, real parts are usually in complex stress state, the only tensile response was analyzed and the basic effect was discussed firstly here. 

### 4.3. Thickness Effect of Alumina Films on Interface Damage and Fracture

The experiments showed that the interface fracture occurred more easily for the thicker coatings due to the competition between the interface tensile and shear stresses [14], how about is the thickness effect of thin films at the same interface tensile case. The topic is intriguing and thus was checked here. The tensile stress-displacement curves for several films with increasing thickness from 100 nm, 10 μm, 100 μm, 300 μm to 500 μm, respectively, were simulated by adjusting corresponding initial stiffness. The total loading displacement was fixed as 400 nm and there was no change in the interface fracture energy for nanocrystalline films.

**Figure 11:**
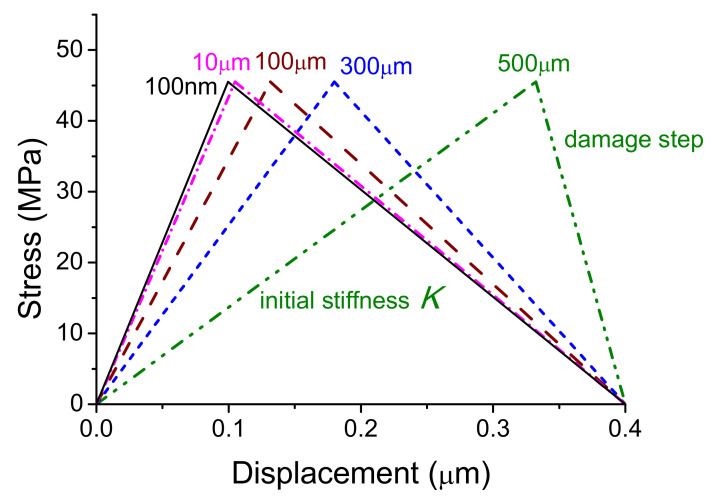
The relationships between tensile stress and total displacement of the systems with different film thickness from 100 nm, 10 μm, 100 μm, 300 μm to 500 μm (from left to right), respectively.

Figure 11 shows that the interface strength keeps a constant with increasing film thickness. However, the damage displacement, corresponding to the peak stress, increases with increasing film thickness as showed in Figure 11, that is, the thicker films research peak stress at the larger displacements, corresponding the smaller initial stiffness. After reaching the interface strength, interface damage speed of the thicker films is faster, meaning that the interface fracture of the thicker films is more catastrophic. Although the phenomenon is not obvious for the film thickness lower than several microns, the thickness-dependent damage speed is in agreement with the previous molecular simulation result [33] (see Figure 3a in Ref. [33]). Understanding this effect of film thickness is helpful to guide a design of film parts.

Note that the initial stiffness *K* of the system, that is, the stiffness at initial step, decreases with increasing film thickness, which can be analyzed based on the series model. According to the series model [33], the total displacement Δ can be expressed as

(9) $$∆=\frac{\sigma }{E_{f}}t_{f}+\frac{\sigma }{E_{s}}t_{s}+\delta$$

where *σ* is same in the film, the substrate and the interface, *E* is the elastic modulus and *t* represents the thickness, the subscripts *f* and *s* denote the film and the substrate, respectively, *δ* is the interface displacement. Equation (9) indicates that the total displacement equals the sum of displacements of the film, substrate and the interface. Therefore, the initial stiffness *K* of the system is as follows

(10) $$K=\frac{1}{\frac{t_{f}}{E_{f}}+\frac{t_{s}}{E_{s}}+\frac{1}{k}}$$

Equation (10) indicates that the system stiffness decreases with increasing film thickness *t_f_* when the modulus and the interface stiffness *k* are fixed, which explains simulation results in the initial step in Figure 11.

The film thickness effect on damage speed can also be analyzed. Substituting Equation (5) into Equation (9), only the function at *δ*^0^ < *δ* < *δ^f^* in Equation (5) corresponding to the interface damage step is considered, the total stress-displacement relation can be obtained at the damage step,

(11) $$\sigma =\frac{\mathrm{MN}}{N-M}k∆-\frac{\left(1+N\right)M}{N-M}\sigma^{0}$$

where *σ*^0^ is the interface strength as shown in Figure 2, M represents material properties and equals $\frac{E_{f}E_{s}}{k\left(E_{f}t_{s}+E_{s}t_{f}\right)}$, N represents interface cohesive relation and is expressed as $\frac{\delta^{0}}{\delta^{f}-\delta^{0}}$ [30]. Therefore, the speed of stress drop in the damage step, that is, the damage speed, can be obtained as

(12) $$\left|\frac{d\sigma }{d∆}\right|=\frac{kN}{1-N/M}$$

Since M decreases with increasing film thickness *t_f_*, the damage speed increases with decreased M based on Equation (12). Therefore, the damage speed of the thicker films is faster as shown in Figure 11. Underlying mechanism is resulted from that elastic energy of the film increases with increasing thickness and needed external force work decreases for interface fracture.

## 5. Conclusions

In summary, 3D finite element models of film/substrate systems under uniaxial tension were developed based on the interface cohesive element and the corresponding interface fracture energy was calculated quantitatively. The detailed Mises stress and damage evolution was demonstrated. After reaching the interface strength, the stress releasing zone localizes to the central film firstly and the interface damage initiates from the central and extends to the circumference up to complete fracture for homogeneous film/substrate structure. For interfaces with radial residual force, the interface strength decreases and the damage initiates from the stress concentration zone. For interfaces with axial residual pressure, the interface strength increases. Damage speed increases with increasing film thickness and underlying mechanism was discussed. Moreover, the interface strength increases for nanocrystalline films compared to that for conventional ones, agreeing with the experimental results. These size and residual stress effects on interface damage and fracture should be considered in the design of film parts.

## Figures and Tables

**Table 1:** The related parameters used in calculating interface free energy.

	*h* (nm)	*S_m_* (Jmol^−1^K^−1^)	*H_m_* (Jmol^−1^)	*T_m_* (K)	*V* (cm^3^mol^−1^)
Al_2_O_3_	0.301 [22]	40.67	111400 [23]	2328 [25]	28.3 [25]
Ni	0.275 [27]	10.12 [28]	17470 [28]	1726 [28]	6.59 [28]

## Data Availability

Data is contained within the article.

## Abbreviations

3D	Three dimensional
ABQUAS	Name of the soft based on finite element method.
C3D8R	Name of the element in ABQUAS, C (entity element), 3D (three dimensional), 8 (number of nodes), R (reduced integral element).
COH3D8	Name of the cohesive element in ABQUAS based on cohesive model, COH (cohesive).
